# GPs’ experience with diagnosis and treatment guidelines for lower UTI in men: a qualitative interview

**DOI:** 10.3399/BJGPO.2023.0254

**Published:** 2024-09-04

**Authors:** Helena Kornfält Isberg, Hedvig Gröndal, Mia Tyrstrup

**Affiliations:** 1 Department of Clinical Sciences, Family Medicine and Community Medicine, Lund University, Malmö, Sweden; 2 Department of Biomedical Sciences and Veterinary Public Health Swedish, University of Agricultural Sciences (SLU), Uppsala, Sweden

**Keywords:** urinary tract infections, primary health care, men's health

## Abstract

**Background:**

The incidence of lower urinary tract infection (LUTI) in men visiting primary health care (PHC) is low. Hence, GPs do not diagnose and treat men with LUTI very often. Previous studies have shown that adherence to treatment guidelines regarding LUTI in men is low. There is limited knowledge concerning why guidelines are not adhered to.

**Aim:**

To gain knowledge on GPs’ experiences and concerns when treating men with LUTI, including their use of clinical guidelines. Furthermore, this study aimed to explore GPs’ knowledge and concern regarding antibiotic resistance.

**Design & setting:**

A qualitative study based on semi-structured interviews with GPs was performed.

**Method:**

15 GPs from seven PHC centres (PHCC) in southern Sweden were interviewed. The interviews were conducted from September 2022 to March 2023. All interviews were audio recorded and transcribed verbatim. A thematic analysis was performed.

**Results:**

GPs had limited experience with and felt less certainty when diagnosing male LUTI. Extended examinations could partially relieve this feeling. GPs were well informed about the Swedish treatment guidelines for LUTI in men and felt safe to treat their patients according to the guidelines. However, GPs also acknowledged that guidelines do not cover all situations and need to be individualised.

**Conclusion:**

Many GPs feel insecure when diagnosing male LUTI. The relatively low prevalence of this condition in PHC could contribute to this feeling. Clear and instructive guidelines regarding both the diagnostic process and adequate antibiotic choices are important to maintain good quality in the management of LUTI in men.

## How this fits in

Male urinary tract infection (UTI) is regarded as more complex and time-consuming to treat than female UTI in clinical practice and guidelines are regarded as being difficult to adhere to. The reasons for this are not yet well understood. This study explored GPs’ concerns regarding diagnosing and treating men with UTI. The knowledge can potentially be used to improve communication about existing clinical guidelines, improve the content of them (for example make them easier to understand), and, by doing so, contribute to a reduction of unnecessary antibiotic prescribing.

## Introduction

Antimicrobial resistance (AMR) is increasing all over the world and has been declared as one of the top 10 global public health threats facing humanity by WHO.^1^ Despite low AMR compared to other countries, continuously increasing levels of AMR have also been seen in Sweden. Over-prescribing of antibiotics is a driver of AMR and various efforts have been established to enhance better and safer antibiotic use. One example is the use of guidelines for the diagnosis and treatment of different infections. Research, however, describes that adherence to guidelines is often lacking and that antibiotics are overused in spite of guidelines. Studies exploring physicians’ management of lower urinary tract infection (LUTI) in men and women indicate that antibiotic prescribing is often non-concordant with guidelines.^2–4^

The incidence of LUTI in men younger than 55 years is low but increases in older men, yet it is still far less prevalent than among women.^5–8^ Thus, most GPs do not treat men with LUTI very often. A qualitative study performed in Ireland suggests that LUTI in men is perceived by GPs as rare and complicated. In the study, GPs expressed ambiguity concerning treatment recommendations and guidelines and instead often relied on clinical experience when prescribing antibiotics.^9^

Treatment guidelines for male urinary tract infection (UTI) vary between countries. In the UK and Ireland, the guidelines recommend trimethoprim or nitrofurantoin as first-line treatment.^10^ The European Association of Urology^11^ recommends that cystitis in men should be classed as a complicated infection and should be treated with antibiotics that penetrate the prostate tissue such as fluoroquinolones or trimethoprim/sulfamethoxazole.

In Sweden, new national treatment guidelines for UTI in outpatient care were published in 2017. Traditionally, male UTIs have been considered complicated infections due to the presumed involvement of the prostatic gland. However, these definitions have been questioned since there is no evidence for the frequency of involvement of the prostate gland in UTI without fever in men. Pivmecillinam or nitrofurantoin for seven days are now the first-choice antibiotic recommendations for men with LUTI in Sweden. A urine culture should always be performed prior to treatment.^12,13^

In Sweden, national guidelines are adopted and published at a county level, often integrated in the internal web system, to make the guidelines easily available for the GPs. Local Strama (The Swedish strategic programme against antibiotic resistance) groups support prescribers in primary care with information on local prescribing patterns, provide training, and promote the implementation of clinical guidelines. The training is based on recurrent meetings at the primary health care centre (PHCC).

The knowledge of why the Swedish guidelines are not adhered to, in relation to UTIs in general and male UTIS in particular, is limited. To our knowledge, there is no former Swedish qualitative study that has explored this matter. The objective of this study was to gain knowledge on GPs’ experiences and concerns when treating men with LUTI, including their trust and use of clinical guidelines. Furthermore, to explore GPs’ knowledge of and concern regarding AMR. The wider aim is to use the knowledge from this study to improve antibiotic prescribing, for example by adjusting guidelines to make them easier for GPs to follow and in this way increase adherence.

## Method

In order to explore GPs’ experiences and concerns regarding male UTI, this study drew on a qualitative approach. Data were collected from clinicians using semi-structured interviews.^14^ We followed the Standards for Reporting Qualitative Research framework.

### Setting and participants

In Sweden, primary care is accessed via local PHCCs, both public and private. They are all financed by the social national insurance system. Acute onset of UTI is predominantly treated in PHC but could be referred to secondary care if needed. The size of the PHCCs varies from a few GPs to up to 20 GPs per PHCC. Training of medical students and junior doctors is part of the daily work at PHCCs. Junior doctors specialising in general medicine (senior house officers, SHO) work independently but under certain supervision/or tutorial for five years. During their five years of training, they also rotate to hospital clinics for shorter work periods. Point of care testing such as c-reactive protein and urine dipsticks are performed at PHCCs. Urine cultures are sent off to the clinical microbiological laboratory at the hospitals. Some blood samples can be analysed directly at the PHCCs, while some are sent to a clinical laboratory to be analysed. Most PHCCs are equipped with a bladder scan to measure residual urine.

Information about the study and an invitation to participate was sent to the managers of 11 low-prescribing PHCCs (159–243 prescriptions of antibiotics per 1000 registered patients per year) and to 12 high-prescribing PHCCs (255–325 prescriptions per 1000 registered patients per year). Three reminders were sent to the 14 PHCCs that did not answer the first invitation. Three PHCCs were not able to participate due to high workload. Seven PHCCs were willing to participate in the study, of which five were low-prescribing and two were high-prescribing. GPs from the seven PHCCs were invited to participate in the interview. A strategic sample of 15 GPs were chosen with regard to age, educational background, and working experience. Included PHCCs were situated in urban as well as rural areas in the county of Skåne, Sweden. No incentive was used.

We used a semi-structured interview schedule with open-ended questions to facilitate the GPs’ own narrative and expressions. The interview schedule (supplementary schedule 1) was developed by two of the researchers (HKI, MT) and designed to address the research questions. The interview schedule was revised after feedback from the third researcher and fellow clinicians. The interview schedule was also adjusted after the first two interviews to create a better structure and flow in the interview. The questions focused on GPs’ experiences of the management of men with LUTI in PHC as well as their opinions regarding treatment guidelines (supplementary guidelines 2). Twelve of the interviews were performed by one of the researchers who is also a GP (HKI) and three were performed by a senior house officer specialising in general medicine. The interviews were voice-recorded and then transcribed verbatim by a professional transcriber.

### Data analysis

All the interviews were read several times by the three researchers, two GPs (HKI, MT) and one social scientist (HG). The authors met regularly to read, code, and discuss the different themes that emerged from the interviews. After having read 15 interviews no new data emerged and the data were found to be saturated.

A thematic analysis was conducted with an inductive approach, to construct and analyse patterns and themes within the data . We used a pragmatic approach to identify and sort meaning units, themes, and codes.^15^ Through analysis and discussions between the three researchers, the codes and meanings were condensed and finally synthesised to descriptions and concepts.^16^

## Results

In total, 15 GPs from seven PHCCs were interviewed individually, 12 at their PHCC, one at home and two via telephone. The interviews took place from September 2022 to March 2023.

Nine of the GPs worked in a PHCC situated in an urban area and six worked in rural areas. The GPs were aged between 28 and 47 years and six (40%) were men. Nine GPs were educated in Sweden and six were educated in other European countries. The GPs’ work experience differed, eight were specialists in general medicine, five were SHOs specialising in general medicine, and the rest were house officers (a qualified doctor practicing under supervision during the first years after graduation). The interviews lasted between 12 and 25 minutes.

The three main themes that emerged from the analysis of the interviews were: 1) managing complexity—GPs reflections on diagnostic concerns related to male UTI, 2) adherence and individualisation—GPs reflections regarding treatment guidelines for male UTI, 3) Guidelines as safety net in relation to AMR—GPs reflections regarding AMR in men with LUTI. Quotes were labelled with each participant’s unique identification number and age.

### Managing complexity—GPs’ reflections on diagnostic concerns related to male UTI

In all the interviews, the GPs describe diagnostics and management of male UTI as difficult and complex. They express uncertainty regarding the diagnosis and mention differential diagnoses such as prostatitis and other underlying conditions:

*'Well, I guess one has to be observant, on urinary retention and some other things and prostate, prostatitis, palpate the testicles,*' (1, 47 years)

*'It is not unusual for men to have urinary problems. But…in those cases they present symptoms with longer duration, with more evacuation problems and such, considering prostatic hypertrophy…but it can also be other things, such as…yes, what can it be…urinary concrements and such that can cause irritative symptoms…*' (13, 36 years)

In several interviews, GPs recall former experiences from complicated cases with unwanted outcomes. Related to this, they express a sense of vigilance and preparedness for possible complications. Thus, previous experiences are described as influencing the way the GPs interpret the patients’ symptoms and decisions regarding the management including antibiotic prescribing:

*'…for some reason, one is more vigilant, even though it is an uncomplicated infection, but somehow it feels like there is a higher risk that it develops into something more complicated*,' (2, 32 years)

UTIs are found to be significantly less common in men than in women and therefore the level of experience around UTI-related symptoms in men is regarded as lower by the interviewed GPs. The GPs describe that this may lead to uncertainty, worries, and even fear. The underlying fear seems to be related to a fear of missing a serious diagnosis resulting in serious complications. They explain that while women often see their GP already knowing their diagnosis, men tend to seek care due to certain symptoms and express concerns without suggesting they are suffering from a UTI:

*'Women seek care with a UTI; men seek care with urinary tract symptoms. But, I guess, it depends on experience, men do not often know what their symptoms stand for. Men have not often experienced it before and do not know anyone who has*,' (2, 32 years)

In this study, there is a large diversity in how GPs address the term uncomplicated and complicated UTI. Some GPs say that a UTI in a man, per definition, is always complicated because of underlying anatomic changes that cause the UTI. Other GPs regard the infection as complicated if there is presence of fever, prostatic engagement, or recurrent infections. Some GPs stated that they never use the terminology of complicated and uncomplicated UTI.

As described above, the GPs in this study find the diagnostic process regarding male UTI as being characterised by uncertainty. However, several GPs also describe that this uncertainty leads to a thorough examination and follow-up. GPs say that they often schedule a longer appointment for men than for women with the same UTI symptoms. All GPs state that when they examine the urine with a urine dipstick and urine culture, almost all GPs mention the importance of investigating if the prostate gland is involved either by clinical examination or a blood sample. More than half of the GPs mentioned that they perform a point of care test (blood sample) to measure C-reactive protein to investigate whether the patients have signs of infection in the blood. Examination via bladder scan to test for residual urine is common, as well as a consultation with or referral to a urologist. Thus, the uncertainty is often described as manageable through extended examinations.

### Adherence and individualisation—GPs reflections regarding treatment guidelines for male UTI

All GPs express that they trust and follow current guidelines for male UTI. They describe that the guidelines are evidence-based and created by specialists in the field. Some GPs state that they adhere to the guidelines and have changed their treatment of male LUTI from broad-spectrum to narrow-spectrum antibiotics as recommended in the 2017-updated guidelines. One GP explained that they initially felt anxiety regarding prescribing narrow-spectrum antibiotics to men with LUTI when the new guidelines were introduced. However, they followed the new guidelines and noticed that they worked:

*'Yes, I do trust them completely… I usually try to be a good doctor and follow them slavishly*,' (2, 32 years)

*'You have to trust Strama’s recommendations regarding antibiotic prescribing, they check resistance patterns and what works and what doesn’t, so you trust that absolutely*,' (14, 28 years)

Digital solutions with support for prescribers to facilitate adherence to guidelines with 'pre-programmed' antibiotic choice and dosages are described as a helpful tool. One doctor jokingly described:

*'I am lazy…that’s why I follow recommendations…it’s the easiest,'* (2, 32 years).

Another similar example follows:

*'It is more time-consuming to prescribe* [antibiotics] *if you don’t use the PMO template* [digital support],' (2, 32 years)

As described in the previous theme the UTI diagnosis in men can pose a challenge and GPs, in this study, describe that if they are not convinced that UTI is the proper diagnosis, they will not feel safe in following the UTI guidelines. One GP explained that due to the complexity, it is not possible to make a flowchart for the diagnosis of male UTIs:

'…*so **if** I feel safe with the diagnosis I trust them…absolutely,'* (3, 47 years)

'…*but according to my experience it is not as obvious, how to diagnose* [a man]*. So I believe the diagnostics I think, but it is also… not as easy to make a flowchart, because the diagnostics…there are so many factors*,' (8, 39 years)

Even though the GPs in this study describe high adherence to guidelines, they also find that guidelines cannot cover all situations but must be individualised. To individualise treatment is described as routine in their daily work:

*'No, guidelines can describe some classic cases, they cannot… as a GP you have to take into account …to your judgement and what is actually happening, you cannot always trust in guidelines…Guidelines are just guidelines after all*,' (15, 42 years)

One question that was addressed during the interviews was how the treatment guidelines could be improved to be more informative and easier to adhere to. Some GPs requested improving the guidelines regarding the diagnostic process. One GP suggested that the guidelines should be clearer in describing when *not* to prescribe antibiotics:

*'…so it is great that we talk about it, because it will be easier to refrain, if you somehow have a guideline. There, it is good with guidelines that indicate that it is ok not to prescribe…then it is easy to refrain*,' (13, 36 years)

A special concern regarding male UTI expressed by the interviewees was the examination of the prostatic gland, which is not mentioned in the guidelines. Several GPs acknowledged that the examination can be uncomfortable for the patient and that this can make them hesitate from performing the examination:

*'But it is always that, should I examine the prostatic gland or not*,' (9, 39 years)

Summing up this section, the GPs describe that they trust and follow guidelines; however, they emphasise that guidelines must be used with some flexibility and adjusted to the individual patient. They also request improved clarity around the diagnostic process and when to abstain from antibiotics.

### Guidelines as a safety net in relation to AMR—GPs reflections regarding AMR in men with LUTI

Despite the fact that AMR is more common in male than in female UTI, the GPs did not express that they felt especially concerned about AMR when treating men with LUTI. Few of the GPs had experience of cases with complicated antibiotic resistance patterns in urine cultures:

'*I don’t have the feeling that it is a problem… but I don’t remember that it has ever been a problem*,' (7, 47 years)

*'My opinion is that men that suffer from LUTI often have uncommon pathogens since they often have predisposing anatomic factors…*' (8, 39 years)

Some of the interviewed GPs say that they don’t think a lot about antibiotic resistance in urine cultures from men in the encounter with patients since they follow guidelines and treat empirically. The question is raised first when the result from the urine culture arrives.

'... *only think of it afterwards if a resistant bacteria is detected otherwise I just follow the recommendations'* (2, 32 years)

In general, most of the interviewed GPs were aware of high resistance numbers to trimethoprim and ciprofloxacin in *E. coli*. They were also aware of the low level of resistance in *E. coli* to the first-choice antibiotics (pivmecillinam and nitrofurantoin) and reported good treatment results with the first-choice antibiotics. Some GPs raised concerns regarding a possible increase in resistance levels to the first-choice antibiotics as prescribing of those drugs becomes more common:

*'...Nitrofurantoin works very well, resistance levels are low and that’s why it is used in the first place*,' (14, 28 years)

'*But now it is only a matter of time until we experience that more and more patients develop resistance against pivmecillinam'* (15 , 42 years)

Most GPs interviewed expressed vague knowledge about the exact antibiotic resistance levels in urine cultures from men in PHC with LUTI. When they reasoned it, they conclude that resistance levels are likely to be higher among men than women since guidelines state that a urine culture should always be performed in a man. Some of the GPs stated that the treatment guidelines are based on antibiotic resistance in *E. coli* and if they follow them, they don’t have to consider resistance patterns prior to each antibiotic prescription. They also explained that their first consideration regarding resistance is made when they check the result from the urine culture.

## Discussion

### Summary

Many of the GPs had limited experience in treating male UTI. The general opinion was that male UTI is more complicated to diagnose than female UTI due to more differential diagnoses in men. Although GPs described the diagnosis of male UTI as characterised by uncertainty and to some extent even worries, they describe the uncertainty as manageable through extended examinations.

All the interviewed GPs were content with the Swedish treatment guidelines and felt safe to treat their patients according to them. However, they emphasised that the guidelines cannot cover all situations, and treatment must be individualised for each patient. Some suggestions to improve the guidelines were also made. The GPs proved to be well-informed about the treatment suggestions for male UTI as presented in the guidelines.

### Strengths and limitations

To our knowledge, no previous Swedish study has explored GPs’ experiences of male UTI and opinions of treatment guidelines and antibiotic resistance. This study gives new knowledge concerning difficulties in the management of lower UTI in men, suggestions for improvement of clinical guidelines, and an insight into the daily work of GPs. A strength of this study was the diversity in the participants’ age, country of education, and work experience. In addition, they work in six different PHCCs situated in both urban and rural areas. This likely contributes to a variety of experiences. The design of the study, with semi-structured interviews and open-ended questions, contributed to multifaceted and detailed information. Three researchers with different scientific backgrounds participated in the analysis of the interviews and this ensured different perspectives in the analysis.

A limitation of the study was that all of the 15 GPs that were interviewed were working within a limited geographic area in Sweden (Skåne). In national surveillance data, Skåne tends to be the county with the highest antibiotic prescribing rates per citizen in Sweden. Moreover, it is possible that the GPs that accepted to participate have a special interest in antibiotic prescribing in general and management of LUTI in men in particular. Therefore, the result might not be representative of all GPs. Moreover, the results may not be generalisable to other countries as PHC, resistance patterns, and treatment guidelines differ between countries. Efforts were made to recruit GPs from other PHCCs but recruitment was difficult. The PHCCs with the highest prescribing rates did not answer the study invitation despite several reminders. We aimed for variation regarding low and high prescribers of antimicrobials, however, the self-selection of PHCCs led to more GPs from low prescribing PHCCs taking part in the study. Furthermore, the mean age of GPs agreeing to the interview was lower than GPs in general in Sweden, which could also influence the results since earlier studies showed that working longer than 10 years as a physician is associated with more inappropriate antibiotic prescribing.^17^

### Comparison with existing literature

In a qualitative study describing patients’ and GPs’ experiences of managing lower urinary tract symptoms in British primary care, the GPs highlighted the difficulties in differentiating between prostate and bladder symptoms to eliminate the possibility of prostate cancer.^18^ While the same problem is addressed also by the GPs in our study, the focus on prostate cancer is not as clearly pointed out. This could be due to the shorter duration of symptoms in relation to an infection as compared to lower urinary tract symptoms, which are often long-lasting. In the British study, GPs described uncertainty and lack of guidance regarding treatment, while in our study GPs expressed great support and trust in the treatment guidelines, at least when they are certain of the diagnosis.

As opposed to an Irish qualitative study on GPs’ attitudes towards diagnosis and treatment of male UTIs, where GPs expressed ambiguity around guidelines,^9^ the GPs in our study felt they had good knowledge of and followed the guidelines. In line with the Irish study, the GPs in our study expressed that they did not treat men with LUTI very often and that they found diagnosing male UTIs more challenging compared to female UTIs. Most of the Swedish GPs did not use the term 'complicated' regarding male UTI and they were not sure of the definition of the term. This contrasts with the Irish study where 9 out of 15 GPs considered a male UTI as complicated.^9^

A systematic review of guidelines for the management of male UTI in primary care shows that the definition and the treatment of UTI in men are imprecise compared to UTI in women. There are different classifications in different countries based on either anatomic or symptomatic classification. Furthermore, recommendations regarding antibiotic choice and length of treatment vary between countries. The term complicated is used differently in different guidelines, which also shows that the UTI diagnosis in men is complex, and even expert groups in different countries have different opinions regarding the management of UTI in men.^19^ Our study showed that GPs often feel insecure in the diagnostic process of UTI in men and often have many differential diagnoses they want to rule out when a man consults with lower urinary tract symptoms.

Evidence to support the new Swedish treatment guideline for male LUTI is lacking as only a few studies have been performed in the field. This is not an issue that seems to concern Swedish GPs as they generally express great trust in the national treatment guidelines. They feel confident that the guidelines are developed by experts in the field. Reasons for this trust could be the effect of the thorough work by Strama in Sweden and the generally high trust in authorities among Swedish inhabitants.^20^

### Implications for practice

Most GPs do not treat UTI in men very often, which could contribute to insecurity in the diagnostic process expressed by many of the interviewed GPs. The insecurity also highlights the importance of clear and instructive guidelines that, in addition to adequate antibiotic choices, provide support in the diagnostic process. However, the study also showed that GPs acknowledge that guidelines cannot cover all patients and situations, but still find them useful and trustworthy.

## Data

The data that support the findings of this study are available from the corresponding author on reasonable request.
